# A new method to measure muscle protein synthesis in humans by endogenously introduced d_9_-leucine and using blood for precursor enrichment determination

**DOI:** 10.14814/phy2.12479

**Published:** 2015-08-04

**Authors:** Lee Tran, Haley Masters, Lori R Roust, Christos S Katsanos

**Affiliations:** 1 Center for Metabolic and Vascular Biology, Arizona State UniversityTempe, Arizona, USA; 2 Mayo Clinic in ArizonaScottsdale, Arizona, USA

**Keywords:** amino acids, d_10_-leucine, fractional synthesis rate, humans, stable isotope tracer

## Abstract

Enrichment from the easily accessible blood amino acid pool is commonly used as precursor enrichment to calculate rates of muscle protein fractional synthesis in relevant human studies in lieu of the less accessible muscle fluid amino acid pool. However, the accuracy of this approach depends largely on the extent to which there is low discrepancy in free amino acid enrichment between blood and muscle. Steady-state gradient (i.e., ratio) of amino acid enrichment between blood and muscle fluid in the basal state and in response to amino acid infusion were determined in five healthy subjects, and in association with two separate tracers: d_9_-leucine, introduced endogenously by the metabolism of d_10_-leucine (i.e., l-[2,3,3,4,5,5,5,6,6,6-^2^H_10_]leucine) infused in blood, and ^13^C_6_-phenylalanine introduced/infused in blood. The blood-to-muscle fluid amino acid enrichment ratio was lower (*P *<* *0.05) for d_9_-leucine compared to ^13^C_6_-phenylalanine both before (1.5 ± 0.1 vs. 2.5 ± 0.1) and during (1.1 ± 0.1 vs. 1.2 ± 0.1) amino acid infusion. Importantly, the decrease in this ratio in association with the amino acid infusion was considerably less for the d_9_-leucine than the ^13^C_6_-phenylalanine (−0.38 ± 0.03 vs. −1.29 ± 0.07; *P *<* *0.05). In conclusion, blood d_9_-leucine enrichment introduced endogenously by intravenous infusion of d_10_-leucine provides a closer estimate of the muscle fluid amino acid enrichment, and its associated changes, than blood phenylalanine enrichment to calculate rates of muscle protein synthesis in humans.

## Introduction

Accurate quantitation of protein dynamics is important when investigating abnormal skeletal muscle protein metabolism in sarcopenia, muscular dystrophies or secondary to metabolic disorders such as obesity and diabetes. Studies interested in quantifying such protein dynamics commonly employ methods based on the in vivo labeling of proteins with stable isotopes of amino acids (i.e., tracers). Choosing a specific stable isotope of an amino acid involves addressing concerns related to cost, availability, and relevance to the question asked. Most importantly, the chosen isotope should be able to provide a valid measure of the variable evaluated and be sensitive enough to detect directional changes under specific experimental manipulations. The most common method to measure fractional synthesis rate of muscle protein(s) in vivo in humans is based on the constant infusion of a labeled amino acid tracer and quantifying the rate of incorporation of this tracer (i.e., precursor) into muscle protein(s) (i.e., product). In this precursor-product model, the rate of tracer incorporation into protein(s) is normalized to the enrichment of the precursor pool (Wolfe and Chinkes 2004).

Although muscle aminoacyl-tRNA is the true precursor for protein synthesis in muscle, enrichment of alternative pools with the amino acid tracer are used in human studies because of the large amount of muscle sample (i.e., >300 mg) currently required to reliably measure the enrichment of aminoacyl-tRNA (Ljungqvist et al. 1997; Chow et al. 2006). Thus, in lieu of the aminoacyl-tRNA, the amino acid enrichment in muscle fluid is largely accepted as an appropriate surrogate precursor enrichment to calculate muscle protein synthesis (Baumann et al. 1994; Ljungqvist et al. 1997). However, continuous monitoring of the enrichment of the free amino acid pool in muscle over the course of an experimental manipulation that interrupts steady-state muscle amino acid metabolism in humans is constrained by the number of muscle biopsy samples that can be reasonably collected under standard clinical experimental settings. Under these circumstances, we (Katsanos et al. 2006, 2009) and others (Koopman et al. 2008, 2009; Luiking et al. 2014) have used an average amino acid enrichment from frequent blood samples collected from the easily accessible blood amino acid pool as precursor enrichment to calculate muscle protein synthesis.

Average blood amino acid enrichment determined from frequent blood samples over time generally reflects fluctuations in the muscle precursor amino acid pool more accurately than the enrichment determined from limited number of muscle biopsy samples. Therefore, using amino acid enrichment in blood rather than muscle fluid as precursor enrichment is recognized as a sensitive tool to detect directional changes in the response of muscle protein synthesis to a physiological challenge. A limitation of using blood amino acid enrichment instead of muscle fluid amino acid enrichment in conjunction with the infusion of standard tracers, however, is the underestimation of the true rate of muscle protein synthesis (Martini et al. 2004; Pennings et al. 2011). The underestimation is largely due to the free amino acid enrichment in blood being higher than that in muscle because of constant dilution of the tracer within muscle from continuous appearance of unlabeled amino acids from tissue protein breakdown. Therefore, and when sampling from the blood, introducing the amino acid tracer directly in muscle (i.e., the tracer appears/is produced metabolically within the muscle) can minimize the discrepancy in the enrichment of free amino acids between blood and muscle.

**Figure 1:**
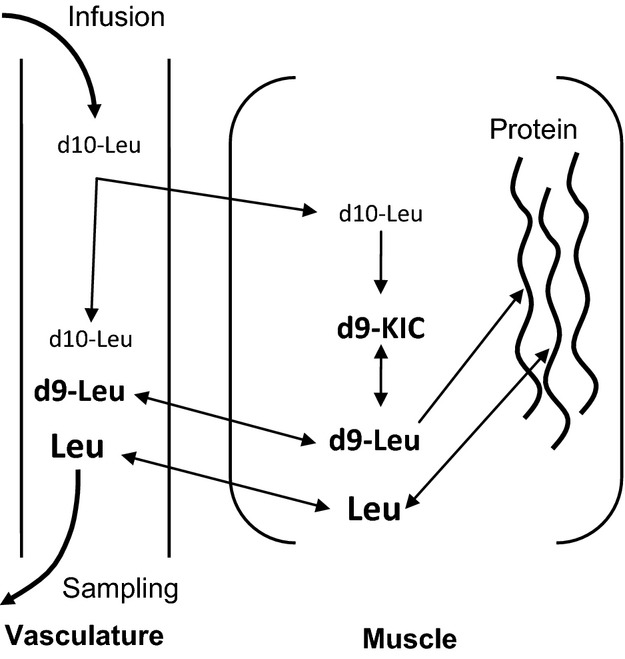
Simplified depiction of intravenously infused d_10_-leucine metabolism in blood and muscle. D_10_-leucine transported into the muscle is transaminated, loses its *α*-carbon deuterium, and via its intermediate *α*-ketoisocaproate (KIC) results in the irreversible formation of d_9_-leucine. D_9_-leucine introduced this way directly into the muscle is available to be mixed with unlabeled leucine coming from muscle protein degradation and blood. Both d_9_-leucine and unlabeled leucine in muscle are then available to be incorporated into muscle protein or transported into the blood in a way that the ratio of labeled-to-unlabeled leucine in blood approximates that of labeled-to-unlabeled leucine in muscle.

A way to introduce a tracer directly into the muscle is by intravenous infusion of l-[2,3,3,4,5,5,5,6,6,6-^2^H_10_]leucine (d10-leucine) that results in endogenously formed d_9_-leucine, because d_10_-leucine through the transamination process in muscle loses its *α*-carbon deuterium and via its intermediate *α*-ketoisocaproate (KIC) results in the irreversible formation of d_9_-leucine (Beynon and Pratt 2005) (Fig.1). Furthermore, and in line with this evidence, preliminary experiments in our laboratory indicated that d_9_-leucine enrichment represents about 88% of the sum of d_9_-leucine + d_10_-leucine enrichment in muscle fluid following intravenous administration of d10-leucine in human subjects. Under these conditions, d_9_-labeled and unlabeled leucine isotopomers from muscle spill over into the blood and can minimize the gradient between blood and muscle fluid d_9_-leucine enrichments. Therefore, introducing the tracer directly into the muscle by the endogenous formation of d_9_-leucine can provide more accurate determination of muscle protein synthesis compared to when the tracer is introduced first into the blood and then transported into the muscle, and when using blood amino acid enrichment as the precursor. It is noted that d_10_-leucine has been previously used to label proteins in cell culture (Pratt et al. 2002; Beynon and Pratt 2005; Dieterich et al. 2006; Jiang and English 2006; Wei et al. 2013) but not human studies.

Under the circumstances discussed above, using d_9_-leucine enrichment in blood following infusion of d_10_-leucine (i.e., “d_10_-to-d_9_-leucine approach”) provides a theoretical advance for the determination of muscle protein synthesis when using blood amino acid enrichment as the precursor for the determination of muscle protein synthesis. However, this approach has not been contrasted to currently well-accepted methodologies. There is a possibility for erroneous results due to the large number of labeled atoms in the tracer relative to the atoms (i.e., ≤6) found in traditional tracers (Zhang et al. 2001). Further, although liquid chromatography tandem mass spectrometry (LC-MS/MS) has emerged as a more precise, sensitive, and reproducible method to evaluate amino acid enrichment relative to traditional mass spectrometry techniques (Zabielski et al. 2013), this method may influence the quantification of amino acid isotope ratio when deuterium-labeled molecules, such as the d_9_-leucine, are measured (Zhang et al. 2001; Rand et al. 2008). Such concerns, if true, can ultimately compromise the ability of the d_10_-to-d_9_-leucine approach to accurately describe differences in muscle protein synthesis.

Therefore, the specific purpose of these experiments was to evaluate the d_10_-to-d_9_-leucine approach in assessing changes in muscle protein synthesis in a circumstance associated with the infusion of amino acids, and by focusing particularly on the gradient in free amino acid enrichment between blood and muscle. Responses associated with this new approach were compared to those obtained using ^13^C_6_-phenylalanine, a well-established and commonly used tracer in human studies of muscle protein metabolism.

## Methods

All the experimental procedures were approved by the Institutional Review Board of the Mayo Clinic. The subjects participating in the experiments were four males and a female (*n* = 5) with an average age of 32 ± 5 years (mean ± SE), body mass 89 ± 10 kg, and body fat (bioelectrical impedance analysis) 29 ± 3%. All subjects were free of disease as determined by the use of a medical history survey, physical examination, resting electrocardiogram, and standard blood and urine tests, and they were not taking any medications or supplements known to affect protein metabolism. Known risks associated with the experimental procedures were explained to each subject prior to obtaining a written consent. Studies were performed in the Clinical Studies Infusion Unit (CSIU) at Mayo Clinic in Arizona.

### Isotopes

l-[2,3,3,4,5,5,5,6,6,6-^2^H_10_]leucine (98% enriched), l-[ring-^13^C_6_]phenylalanine (99% enriched), l-[U-^13^C_9_-^15^N]phenylalanine (U-^13^C_9_, 97–99% and ^15^N, 97–99% enriched), and l-[U-^13^C_6_]leucine (97–99% enriched) were purchased from Cambridge Isotope Laboratories (Andover, MA). All isotopes were tested prior to shipping for sterility and pyrogenicity by the manufacturer. l-[2,3,3,4,5,5,5,6,6,6-^2^H_10_]leucine and l-[ring-^13^C_6_]phenylalanine were used for infusion and were dissolved in normal saline (0.9% NaCl) the morning of the experiment by the pharmacy at Mayo Clinic in Arizona. l-[2,3,3,4,5,5,5,6,6,6-^2^H_10_]leucine undergoes transamination within the cells losing the *α*-carbon deuterium resulting in the irreversible formation of d_9_-leucine, and as depicted Figure1. Thus, d_9_-leucine was used as the tracer to describe muscle protein metabolism.

### Experimental protocol

Subjects were admitted to the CSIU at 6:30 am the morning of the study, and after an overnight fast (i.e., consumed nothing except water after 10:00 pm). Subjects were also instructed to avoid any form of exercise for the 3-day period preceding the study, and compliance was verbally verified the morning of the study. At ∼7:30 am, an 18-gauge polyethylene catheter was inserted into an antecubital vein of an arm for infusion of the amino acid tracers. A separate catheter was inserted in a retrograde fashion in a dorsal hand vein of the opposite arm for the collection of “arterialized” blood samples using the heated-hand technique.

**Figure 2:**
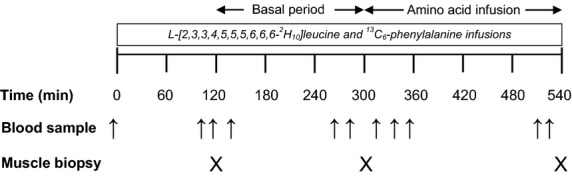
Experimental protocol depicting the basal period and the period associated with the infusion of amino acids. The leucine and phenylalanine tracers specified were infused throughout the experimental protocol. Blood and muscle samples were collected at the time points depicted.

The experimental protocol was composed of two periods, one describing the response of muscle protein synthesis during basal/postabsorptive conditions (i.e., basal) and the other the same response during intravenous amino acid infusion to stimulate muscle protein synthesis (i.e., AA; Fig.2). Infusion of the amino acid tracers initiating the experiments was started after the collection of blood samples for the measurement of background amino acid enrichments (∼8:00 am), and continued for 300 mins in association with the basal period. The infusion rates of the tracers during the basal period were as follows: l-[2,3,3,4,5,5,5,6,6,6-^2^H_10_]leucine, 0.15 *μ*mol kg/FFM/min (priming dose, 9.0 *μ*mol kg/FFM); l-[ring-^13^C_6_]phenylalanine, 0.06 *μ*mol kg/FFM min (priming dose, 3.0 *μ*mol kg/FFM). At 300 min, an amino acid mixture (15% Clinisol; Baxter Healthcare Corporation, Deerfield, IL) was started at 4 mg kg/FFM/min (priming dose, 82 mg kg/FFM), and was continued for 240 min (i.e., 540 min from the beginning of the experiments). During the amino acid infusion, the infusion rates for the tracers were increased as follows to account for dilution of the tracers by the exogenous amino acids: l-[2,3,3,4,5,5,5,6,6,6-^2^H_10_]leucine, 0.29 *μ*mol kg/FFM/min (priming dose, 2.6 *μ*mol kg/FFM); l-[ring-^13^C_6_]phenylalanine, 0.32 *μ*mol kg/FFM/min (priming dose, 2.1 *μ*mol kg/FFM). The rate of infusion of the amino acid mixture was chosen based on previous evidence indicating that this rate is sufficient to stimulate muscle protein synthesis in healthy humans (Bohe et al. 2003).

Three muscle biopsies (30–40 mg) were collected from the *vastus lateralis* using the Bergstrom needle technique, and at the time points depicted in Figure2. After removing visible fat and connective tissue, the muscle was rinsed with ice-cold saline to remove blood and blotted dry prior to freezing immediately in liquid nitrogen and subsequently stored at −80°C. Samples to determine d_9_-leucine and ^13^C_6_-phenylalanine enrichments in the blood in the basal period and during amino acid infusion were drawn at the time points depicted in Figure2.

### Sample processing

Muscle was processed for muscle fluid and mixed-muscle protein amino acid enrichments using standard procedures (Katsanos et al. 2005). The details of these procedures are as follows: after weighing approximately 15 mg of muscle tissue, 500 *μ*L of 5% sulfosalicylic acid (SSA) was added to precipitate the muscle proteins. An internal standard (2 *μ*L/mg wet tissue) containing l-[U-^13^C_6_]leucine (5.6 *μ*mol/L) and l-[U-^13^C_9_-^15^N]phenylalanine (2.6 *μ*mol/L) were added to measure muscle fluid free leucine and phenylalanine concentrations. The muscle was homogenized and centrifuged at 2500 ***g*** for 45 min at 4°C, and the supernatant was collected. About 500 *μ*L of 5% SSA was added again, and after the procedure was repeated one more time, the pooled muscle fluid was stored at −80°C. The resulting muscle pellet was first washed with 500 *μ*L of 5% SSA, then 1 mL of ethanol, and lastly with 1 mL of ethyl ether, and placed in an oven overnight at 50°C and until dry. Proteins in the muscle pellet were hydrolyzed the next day with 6 N HCl and by placing at 110°C for 24 h. Muscle fluid corresponding to 10 mg of wet tissue weight and protein hydrolysate corresponding to 1 mg of dry tissue weight were passed through cation-exchange column (AG 50W-8x 200–400-mesh; Bio-Rad Laboratories, Inc., Hercules, CA) to isolate/purify the amino acids. Prior to the addition of the samples, the columns were conditioned with 3 mL of 2 N NH_4_OH and 3 mL of 1 N HCl, and the amino acids were eluted with 8 mL of 2 N NH_4_OH.

Collected blood samples were transferred into preweighed tubes containing 1 mL of 15% SSA and 100 *μ*L/mL blood of l-[U-^13^C_6_]leucine (5.6 *μ*mol/L) and l-[U-^13^C_9_-^15^N]phenylalanine (2.6 *μ*mol/L) to determine blood leucine and phenylalanine concentrations, and the samples were mixed well. The difference in tube weight before and after the addition of the blood sample was recorded as the blood sample weight. The blood/SSA mixture was centrifuged at 2500 ***g*** for 15 min at 4°C, and the supernatant was collected. Cation-exchange columns (AG 50W-8x 100–200-mesh; Bio-Rad Laboratories, Inc.) were used to isolate the blood amino acids and after conditioning of the columns as described above for muscle. 500 *μ*L of the blood/SSA mixture supernatant was added to each column, and the amino acids were eluted using 8 mL of 2 N NH_4_OH.

### Mass spectrometry analyses

The isotopic enrichment of amino acids was measured by LC-MS/MS, using the isobutyl ester derivative of the amino acids, and expressed as molar percent excess (MPE). Selected reaction monitoring was applied and transitions of *m*/*z* 220 → 120, 226 → 126 and 230 → 130 were used for the quantification of the m + 0, m + 6 and m + 10 phenylalanine isotopes, respectively, in the blood and muscle fluid. For the determination of mixed-muscle protein phenylalanine isotopic enrichment, transitions of *m*/*z* 222 → 122 (m + 2) and 226 → 126 (m + 6) were quantified and m + 6/m + 0 enrichment was calculated using a calibration curve (i.e., MPE vs. m + 6/m + 2). Details pertaining to the determination of phenylalanine enrichment using LC-MS/MS have been previously described (Zabielski et al. 2013).

The isotopic enrichment of leucine was measured in the same LC-MS/MS run as that for the isotopic enrichment of phenylalanine, with selected reaction monitoring applied for transitions of *m*/*z* 188 → 86, 194 → 92, and 197 → 95 for the quantification of m + 0, m + 6, and m + 9 leucine isotopes, respectively, in the blood and muscle fluid. For the determination of mixed-muscle protein leucine isotopic enrichment, transitions of *m*/*z* 190 → 88 (m + 2) and 197 → 95 (m + 9) were quantified and m + 9/m + 0 enrichment was calculated using a corresponding calibration curve. (i.e., MPE vs. m + 9/m + 2).

### Calculations

Leucine and phenylalanine concentrations in the blood and muscle fluid were quantified from calibration curves using the l-[U-^13^C_6_]leucine and l-[U-^13^C_9_-^15^N]phenylalanine internal standards. The measured values (i.e., nmol) were adjusted to the volume of blood and muscle fluid processed.

The gradient between blood and muscle amino acid enrichment was calculated for each subject as the ratio of blood MPE-to-muscle fluid MPE. Fractional synthesis rate (FSR; % per hour) of mixed-muscle protein was calculated as previously described (Katsanos et al. 2009):

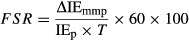
where ΔIE_mmp_ defines the increment in mixed-muscle protein leucine or phenylalanine isotopic enrichment (i.e., MPE) between two biopsies, IE_p_ is the corresponding precursor isotopic enrichment (in either blood or muscle fluid), and *T* is the time interval (min) between biopsies (the factors 60 and 100 are used to express the FSR values in %/hour). In the basal period, IE_p_ was calculated as the average of the enrichment values measured in either the five collected samples in the case of blood or the two collected samples in the case of muscle fluid. In the amino acid infusion period, IE_p_ was calculated as the average of the enrichment values measured in the five collected samples in the case of blood, while the isotopic enrichment in muscle fluid in the last biopsy sample was used for muscle IE_p_ for both leucine and phenylalanine tracers. This is because isotopic enrichment in muscle at the end of the amino acid infusion period is closer to the isotopic steady-state enrichment expected in the course of the amino acid infusion, and given that the amino acid infusion resulted in an initial perturbation in muscle amino acid metabolism and isotopic enrichment in the period immediately after the initiation of the infusion of the amino acid mixture.

### Statistical analysis

Data were analyzed using two-way analyses of variance (ANOVA) to evaluate main effects for factors time (i.e., Basal vs. AA infusion) and tracer (i.e., d_9_-leucine vs. ^13^C_6_-phenylalanine) on the parameters of interest. Differences between the two tracers were compared using paired *t* tests. Changes in parameters of interest over time were analyzed using one-way ANOVA, followed by Tukey’s post hoc tests when significant differences were detected. A *P* value less than 0.05 was considered statistically significant. Experimental data are summarized as means ± SE. Data were analyzed using the Minitab® 16 statistical software (Minitab Inc., State College, PA).

## Results

### Blood and muscle amino acid concentrations and enrichments

**Figure 3:**
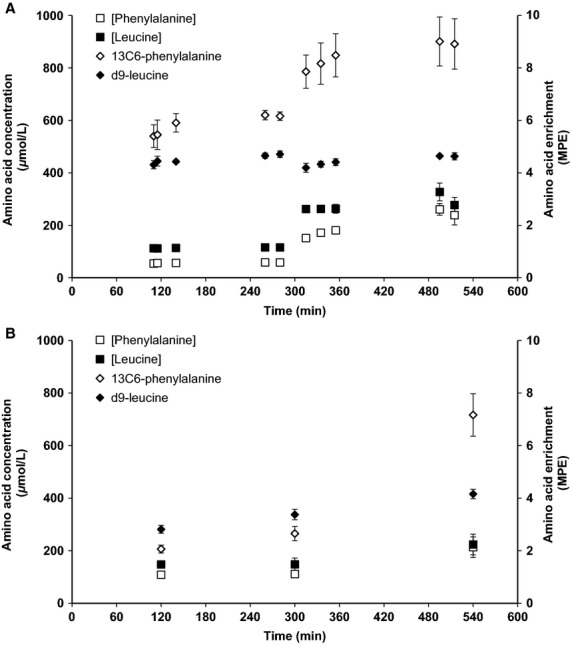
Leucine and phenylalanine concentrations and their respective enrichments in blood (A) and muscle fluid (B) in the course of the experimental protocol. MPE, molar percent excess.

Figure3A shows the blood leucine and phenylalanine concentrations over time. The average blood leucine and phenylalanine concentrations increased during the AA period (*P *<* *0.05; Table1). Average values for blood d_9_-leucine and ^13^C_6_-phenylalanine enrichments (also shown in Fig.3A) fluctuated between 6% and 12%, respectively in the basal period, and between 7% and 9%, respectively during the AA period. Average d_9_-leucine and ^13^C_6_-phenylalanine enrichments in blood were not different between the basal and AA periods (*P *>* *0.05; Table1).

**Table 1:** Amino acid concentrations and enrichments in blood and muscle fluid in the Basal period (Basal) and during amino acid infusion (AA)

	Basal	AA
Blood		
Leucine, *μ*mol/L	114 ± 7	280 ± 14#
Phenylalanine, *μ*mol/L	57 ± 5	204 ± 19#
d_9_-leucine, MPE	4.5 ± 0.1	4.4 ± 0.1
^13^C_6_-phenylalanine, MPE	5.8 ± 0.3	8.5 ± 0.8
Muscle fluid		
Leucine, *μ*mol/L	140 ± 19	223 ± 39*
Phenylalanine, *μ*mol/L	106 ± 11	214 ± 39*
d_9_-leucine, MPE	3.1 ± 0.1	4.2 ± 0.2#
^13^C_6_-phenylalanine, MPE	2.4 ± 0.2	7.2 ± 0.8#
Blood MPE/muscle fluid MPE		
d_9_-leucine	1.5 ± 0.1	1.1 ± 0.1#
^13^C_6_-phenyalanine	2.5 ± 0.1†	1.2 ± 0.1#†

Values are means ± SE; MPE, molar percent excess.

Basal versus AA infusion:

# *P* < 0.01

* *P *≤* *0.05.

D_9_-leucine versus 13C_6_-phenylalanine:

† *P* < 0.01.

Figure3B shows the muscle fluid leucine and phenylalanine concentrations over time. The average muscle fluid leucine and phenylalanine concentrations were higher in the last biopsy (i.e., during the amino acid infusion) when compared to the corresponding average concentrations at basal (*P *<* *0.05; Table1). Muscle fluid d_9_-leucine and ^13^C_6_-phenylalanine enrichments were not different between the first and second biopsy at basal (*P *>* *0.05). However, the average muscle fluid enrichments for both d_9_-leucine and ^13^C_6_-phenylalanine were higher in the last biopsy when compared to the corresponding average enrichments in muscle fluid at basal (*P *<* *0.05; Table1).

One-way ANOVA indicated that mixed-muscle protein isotopic enrichments differed across the three muscle biopsies for both d_9_-leucine (*P *<* *0.05) and ^13^C_6_-phenylalanine (*P *<* *0.05). Post hoc comparisons for d_9_-leucine indicated significant differences (*P *<* *0.05) for mixed-muscle protein enrichment across all three muscle biopsies (MPE; biopsy 1, 0.0086 ± 0.0009; biopsy 2, 0.0175 ± 0.0011; biopsy 3, 0.0391 ± 0.0012). Post hoc pairwise comparisons for ^13^C_6_-phenylalanine indicated significant differences for mixed-muscle protein enrichment between the third (MPE, 0.0424 ± 0.0035) and both the first (MPE, 0.0037 ± 0.0009) and second (MPE, 0.0114 ± 0.0007) muscle biopsies (*P *<* *0.05), but not between the first and second muscle biopsies (*P *>* *0.05).

### Gradient between blood and muscle amino acid enrichment

**Figure 4:**
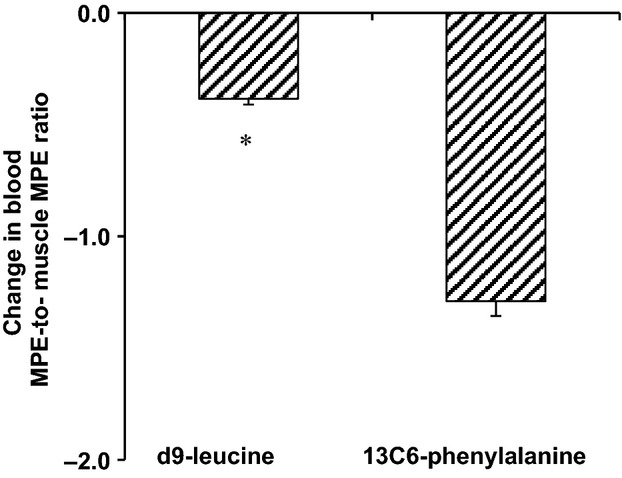
Change from basal in the ratio of blood-to-muscle fluid amino acid enrichment as a result of the intravenous infusion of amino acids (i.e., delta ratio between basal and amino acid infusion). The ratios for both d_9_-leucine and ^13^C_6_-phenylalanine tracers were calculated by dividing the amino acid enrichment (i.e., molar percent excess [MPE]) in blood by the corresponding amino acid enrichment in muscle fluid. *P < 0.05.

Two-way ANOVA indicated significant main effects for both time (*P *<* *0.05) and tracer (*P *<* *0.05) with respect to the blood amino acid enrichment-to-muscle fluid amino acid enrichment ratio (i.e., blood amino acid MPE/muscle fluid amino acid MPE). This gradient in amino acid enrichment was higher for the ^13^C_6_-phenylalanine tracer than the d_9_-leucine tracer in the basal period and, although it decreased for both ^13^C_6_-phenylalanine and d_9_-leucine, it remained higher for the ^13^C_6_-phenylalanine tracer during the AA infusion (Table1). Accordingly, the change in the blood amino acid enrichment-to-muscle fluid amino acid enrichment ratio resulting from the AA infusion was considerably less for the d_9_-leucine when compared to that for the ^13^C_6_-phenylalanine (Fig.4).

### Protein fractional synthesis rate

**Figure 5:**
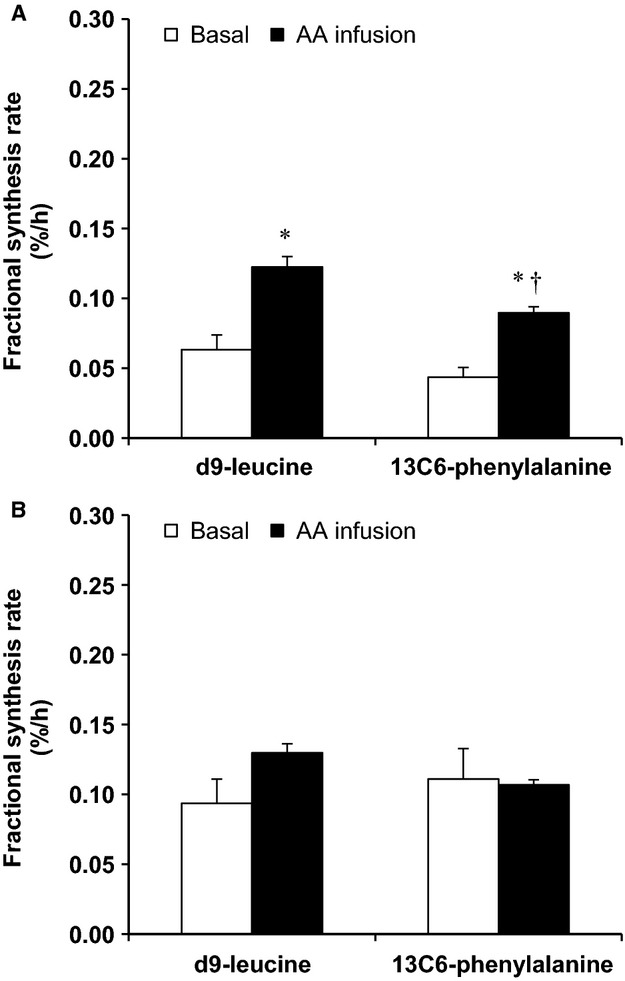
Fractional synthesis rate in the basal period (Basal) and during intravenous infusion of amino acids (AA infusion) determined using either endogenously introduced d_9_-leucine tracer (via intravenous infusion of d_10_-leucine) or intravenously introduced ^13^C_6_-phenylalanine tracer, and using the blood (A) or muscle (B) amino acid enrichment as the precursor amino acid enrichment. *P <* *0.05, basal versus AA infusion; ^†^*P* < 0.05, d_9_-leucine versus ^13^C_6_-phenylalanine.

Two-way ANOVA analysis indicated significant main effects for time (i.e., basal vs. AA; *P *<* *0.05) as well as tracer (*P *<* *0.05) when blood amino acid enrichment was used as precursor enrichment for the calculation of muscle protein FSR. The calculated muscle protein FSR showed significant increase as a result of the amino acid infusion when either blood d_9_-leucine or blood ^13^C_6_-phenylalanine was used as tracer (Fig.5A). Further, as also shown in Figure5A, muscle protein FSR calculated using the blood ^13^C_6_-phenylalanine enrichment was significantly lower than that using the blood d_9_-leucine enrichment in the AA period. When muscle fluid amino acid enrichment was used as precursor enrichment for the calculation of muscle protein FSR, two-way ANOVA showed no significant main effects for either time or tracer (*P *>* *0.05; Fig.5B).

## Discussion

The objective of the present study was to compare a novel approach associated with the measurement of d_9_-leucine enrichment introduced endogenously by intravenous infusion of d_10_-leucine with an approach that is based on a traditional phenylalanine tracer in minimizing the gradient between blood and muscle fluid free amino acid enrichments in the basal state as well as during amino acid infusion. This is because blood is commonly used, instead of the not so easily accessible muscle fluid pool, for precursor amino acid enrichment determination in human muscle protein synthesis studies. Under such a circumstance, blood d_9_-leucine enrichment provides a closer estimate of the muscle fluid amino acid enrichment than blood phenylalanine enrichment when calculating rates of muscle protein synthesis in humans.

The ability of the d_9_-leucine enrichment in blood to more closely reflect the d_9_-leucine enrichment in muscle fluid, when compared to the corresponding phenylalanine enrichments in blood and muscle fluid, is attributed to the introduction of d_9_-leucine directly into the muscle. As opposed to the introduction of ^13^C_6_-phenylalanine first into the blood and its subsequent dilution by continuous release of unlabeled phenylalanine from muscle protein breakdown when it enters the muscle, the difference in amino acid enrichments between the blood and muscle free amino acid pools is minimized using the leucine tracer because both the d_9_-leucine and the unlabeled leucine are introduced first within the muscle free amino acid pool before spilling over into the blood.

The increase in the rate of ^13^C_6_-phenylalanine infusion in blood during the amino acid infusion, which was done in an effort to maintain the ^13^C_6_-phenylalanine enrichment constant following the dilution of the tracer by the infused unlabeled phenylalanine, increased the ^13^C_6_-phenylalanine free amino acid enrichment in muscle in the last biopsy relative to that in basal. This resulted in a decrease in the blood-to-muscle fluid ratio of ^13^C_6_-phenylalanine enrichment during the amino acid infusion relative to that in the basal period (Table1). However, and although the same phenomenon was observed for the leucine tracer, the blood enrichment-to-muscle fluid enrichment ratio during the amino acid infusion decreased considerably less for the d_9_-leucine enrichment when compared to the ratio for the phenylalanine enrichment (Fig.4). This indicates that during transitioning from the basal state to the amino acid infusion state, d_9_-leucine enrichment in blood describes corresponding changes in amino acid enrichment in muscle fluid more closely than the phenylalanine enrichment in blood. Lower perturbation of the d_9_-leucine enrichment in muscle following the amino acid infusion suggests that when blood amino acid enrichment is used as the precursor amino acid enrichment, the d_10_-to-d_9_-leucine approach provides more accurate methodology to measure changes in muscle protein synthesis in humans than that associated with the use of a phenylalanine tracer.

Chinkes et al. (1996) have previously described a similar approach, where labeled leucine (i.e., ^13^C-leucine) is introduced within tissue via intravenous infusion of labeled KIC (i.e., *α*-[1-^13^C]ketoisocaproate). Although both the KIC-infusion approach and the d_10_-to-d_9_-leucine approach share in part some of the same metabolic pathways, it is not necessary that infused KIC and infused leucine are metabolized exactly the same way in tissues. Furthermore, the muscle (i.e., endogenous) free leucine pool is enriched with leucine, rather than KIC, from the breakdown of muscle proteins, as well as amino acids from blood. An additional advantage of the d_9_-leucine tracer over the ^13^C-leucine tracer is its large mass that allows clear separation of isotopic envelopes corresponding to labeled and unlabeled peptides, thus ensuring accurate tandem mass spectrometry-quantification of labeled and unlabeled peptides when determining enrichment at single muscle protein level (Everman et al. 2011). Finally, as opposed to the KIC method, the method described here was evaluated also in association with a perturbation of muscle free amino acids, including leucine, induced by the amino acid infusion which disrupts basal steady-state KIC/leucine metabolism.

Stable isotope enrichment of the amino acids in the present study was measured using LC-MS/MS instead of traditional gas chromatography (GC)-MS, given that LC-MS/MS is gaining rapid popularity in the field of stable isotope analysis of amino acids (Meesters et al. 2009; Zabielski et al. 2013). However, concerns have been raised with respect to the use and analysis of deuterium-enriched molecules because of possible hydrogen-deuterium exchange effects when using LC-MS/MS (Zhang et al. 2001; Rand et al. 2008). With respect to that, the rate of muscle protein synthesis at basal and the increase in protein synthesis due to the amino acid infusion determined using the blood d_9_-leucine enrichment were largely comparable to those determined using the blood phenylalanine enrichment, serving as control in the present study. It is noted that the current approach cannot be evaluated with a phenylalanine tracer because phenylalanine is not metabolized in muscle (Goldberg and Odessey 1972), and in a way that the phenylalanine tracer can serve as endogenously introduced tracer. Although responses using the d_9_-leucine tracer were compared to those of a phenylalanine tracer, because phenylalanine is currently the best established tracer in studies of muscle protein metabolism, it is also noted, that the tracer itself (i.e., leucine vs. phenylalanine) does not influence the ability to detect changes in muscle protein fractional synthesis rate (Harber et al. 2011).

When the reliable amino acid precursor enrichment originating from the multiple blood samples between the biopsies was used for the calculation of muscle protein synthesis, a higher rate of FSR associated with the leucine tracer compared to the phenylalanine tracer (i.e., ANOVA results for tracer main effect) is in line with similar findings reported previously using d_3_-leucine and ^13^C_6_-phenylalanine tracers (Smith et al. 2007). Because the amino acid enrichment in this later report (Smith et al. 2007) was measured using GC-MS, comparable responses in the calculated FSR values when using the leucine and the phenylalanine tracers between that report and the present study suggest that the leucine tracer itself, as well as the type of mass spectrometer employed to analyze d_9_-leucine enrichment (i.e., LC-MS/MS vs. GC-MS), do not have any effects on the evaluation of muscle protein synthesis. Such lack of analytical (i.e., sample processing/mass spectrometry-related) effects in the present study was also expected because the deuteriums in the d_9_-leucine are all bonded to carbon atoms that do not exchange (Whitelegge 2009; Rudowska et al. 2012).

The methodological approach investigated in the present study is of particular practical importance (the cost of the d_10_-leucine tracer is largely comparable to that of ^13^C_6_-phenylalanine) when blood is sought as an alternative to muscle fluid to describe precursor amino acid enrichment for muscle protein synthesis. Because of the low gradient between blood and muscle free amino acid enrichment with the endogenously introduced d_9_-leucine tracer, blood d_9_-leucine provides a better estimate of the precursor amino acid enrichment than blood labeled phenylalanine assuming that muscle fluid amino acid enrichment is an appropriate surrogate of aminoacyl-tRNA enrichment (Baumann et al. 1994; Ljungqvist et al. 1997). Furthermore, low gradient between blood and muscle free d_9_-leucine enrichment overcomes the practical limitation for sampling simultaneously from two separate free amino acid pools when considering some evidence that enrichment of tRNA with labeled amino acids is somewhere between the enrichments of free amino acids measured in blood and muscle fluid (Watt et al. 1991; Young et al. 1994). Interestingly, when an average value between arterialized blood and muscle fluid enrichments was used as precursor amino acid enrichment for the calculation of the rate of muscle protein synthesis, protein synthesis at basal in the present study (0.08 ± 0.01%/h) was similar (0.09 ± 0.02%/h) to the only human study we are aware of using leucyl-tRNA for the calculation of muscle protein synthesis (Watt et al. 1991). Also, lower perturbation of the blood-to-muscle fluid d_9_-leucine enrichment when compared to that of the ^13^C_6_-phenylalanine enrichment after the amino acid infusion ensures reduced error in evaluating changes in muscle protein synthesis with the d_9_-leucine tracer under similar circumstances and when using blood amino acid enrichment as the precursor enrichment. The low gradient between blood and muscle fluid amino acid enrichment with the d_10_-to-d_9_-leucine approach provides a benefit similar to that of the flooding dose technique to measure muscle protein synthesis and where the goal is to minimize the difference in amino acid enrichment between blood and muscle fluid (Garlick et al. 1989). However, the approach described in the present study provides the additional benefit of circumventing the need to increase the blood-labeled amino acid more than tracer amounts when considering that a flooding dose of amino acids stimulates muscle proteins synthesis (Smith et al. 1992).

In conclusion, the strategic use of d_10_-leucine to introduce endogenously d_9_-leucine tracer minimizes the blood-to-muscle fluid gradient in amino acid (i.e., d_9_-leucine) enrichment in the basal state and results in minimal disruption of this gradient during experimental manipulations, such as amino acid infusion, when compared to the traditional infusion of a phenylalanine tracer. The d_10_-to-d_9_-leucine approach describes a novel pragmatic method to measure muscle protein synthesis in humans when the easily accessible blood amino acid pool is sought as an alternative to the muscle fluid amino acid pool to describe precursor enrichment.
